# Evolutionary Engineering in Chemostat Cultures for Improved Maltotriose Fermentation Kinetics in *Saccharomyces pastorianus* Lager Brewing Yeast

**DOI:** 10.3389/fmicb.2017.01690

**Published:** 2017-09-08

**Authors:** Anja Brickwedde, Marcel van den Broek, Jan-Maarten A. Geertman, Frederico Magalhães, Niels G. A. Kuijpers, Brian Gibson, Jack T. Pronk, Jean-Marc G. Daran

**Affiliations:** ^1^Department of Biotechnology, Delft University of Technology Delft, Netherlands; ^2^HEINEKEN Supply Chain, Global Innovation and Research Zoeterwoude, Netherlands; ^3^VTT Technical Research Centre of Finland Ltd. Espoo, Finland

**Keywords:** brewing, *Sacchromyces pastorianus*, evolutionary engineering, chemostat, maltose, maltotriose consumption rate, transport

## Abstract

The lager brewing yeast *Saccharomyces pastorianus*, an interspecies hybrid of *S. eubayanus* and *S. cerevisiae*, ferments maltotriose, maltose, sucrose, glucose and fructose in wort to ethanol and carbon dioxide. Complete and timely conversion (“attenuation”) of maltotriose by industrial *S. pastorianus* strains is a key requirement for process intensification. This study explores a new evolutionary engineering strategy for improving maltotriose fermentation kinetics. Prolonged carbon-limited, anaerobic chemostat cultivation of the reference strain *S. pastorianus* CBS1483 on a maltotriose-enriched sugar mixture was used to select for spontaneous mutants with improved affinity for maltotriose. Evolved populations exhibited an up to 5-fold lower residual maltotriose concentration and a higher ethanol concentration than the parental strain. Uptake studies with ^14^C-labeled sugars revealed an up to 4.75-fold higher transport capacity for maltotriose in evolved strains. In laboratory batch cultures on wort, evolved strains showed improved attenuation and higher ethanol concentrations. These improvements were also observed in pilot fermentations at 1,000-L scale with high-gravity wort. Although the evolved strain exhibited multiple chromosomal copy number changes, analysis of beer made from pilot fermentations showed no negative effects on flavor compound profiles. These results demonstrate the potential of evolutionary engineering for strain improvement of hybrid, alloploid brewing strains.

## Introduction

Over the last decades, the global beer industry has grown to reach a volume of 193 billion liters (data for 2015, https://www.statista.com). Lager beer accounts for 89% of this volume, making it the most-produced fermented beverage. The microbial work horse of lager fermentation is *Saccharomyces pastorianus*, a natural hybrid of *Saccharomyces cerevisiae* and *Saccharomyces eubayanus* (Nakao et al., 2009; Libkind et al., 2011) that has been domesticated in Europe since the late Middle Ages (Meussdoerffer, 2009). Two main *S. pastorianus* lineages emerged from this domestication, commonly referred to as “Saaz” (Group 1) and “Frohberg” (Group 2) strains (Dunn and Sherlock, 2008). These groups display distinctive phenotypic characteristics. Saaz yeasts generally exhibit a higher cold tolerance and stronger tendency to flocculate, while Frohberg strains exhibit faster fermentation and maltotriose conversion (Gibson et al., 2013; Gibson and Liti, 2015). These characteristics coincide with differences in their genome composition. In Saaz strains, a sizeable fraction of the *S. cerevisiae* subgenome (e.g., chromosomes (CHR) VI and VIII as well as parts of IV, XIII, and XV) is absent, whereas modern industrial Frohberg yeasts retain a near-complete set of chromosomes from both parents (Hewitt et al., 2014; Walther et al., 2014; van den Broek et al., 2015). Origins of these two lineages is still a matter of debate, some studies have advocated for two different hybridization histories (Dunn and Sherlock, 2008; Nguyen et al., 2011; Okuno et al., 2016) whereas others privileged a common primary hybridization event followed by divergent evolutionary paths (Okuno et al., 2016).

The growing popularity of lager beer provided the brewing industry with incentives to intensify the industrial fermentation process (Casey et al., 1983, 1984). This intensification was primarily achieved by increasing the gravity of wort. Gravity, in the context of brewing, indicates the density of the wort relative to that of water and predominantly depends on the sugar content (He et al., 2014). Current industrial lager fermentations are performed at concentrations of fermentable sugars ranging from 150 to 200 g·L^−1^ (16 to 20° Plato gravity equivalent). High-gravity fermentation improves process economics as well as sustainability (Stewart et al., 2013). Malted barley wort, the substrate for lager brewing, contains five main fermentable sugars: the monosaccharides glucose and fructose, the disaccharides maltose and sucrose and the trisaccharide maltotriose. Wort dextrins, which are glucose chains with more than three moieties, contribute to wort gravity but cannot be fermented by *S. pastorianus* strains (Sills and Stewart, 1982).

Wort fermentation by *S. pastorianus* strains is usually initiated by a fast, preferential consumption of glucose and fructose. In *Saccharomyces* yeasts, these sugars are transported across the plasma membrane by a large set of hexose facilitator (HXT) transporters. The genome of the laboratory model *S. cerevisiae*, encompasses 23 *HXT* genes (Wieczorke et al., 1999). Hxt transporters in *S. eubayanus*, the other contributor to the hybrid genome of *S. pastorianus*, have not yet been characterized in detail. However, its genome harbors *nine* genes annotated as *HXT* orthologs (Baker et al., 2015; Hebly et al., 2015). In addition to these energy-independent hexose facilitators, a fructose/H^+^ symporter, inherited from *S. eubayanus*, has been identified in *S. pastorianus*, but not in *S. cerevisiae* (Goncalves et al., 2000; Pengelly and Wheals, 2013). Maltose and maltotriose represent over 70% of the total sugar content of wort (Otter and Taylor, 1967). These oligosaccharides are typically taken up by *S. pastorianus* strains after depletion of glucose and fructose (Gibson et al., 2008; He et al., 2014).

In *Saccharomyces* yeasts, maltose is transported across the plasma membrane by proton symporters (Serrano, 1977; van Leeuwen et al., 1992; Weusthuis et al., 1993; van den Broek et al., 1994). Maltose-proton symport is energized by the plasma-membrane proton-motive force, which is maintained by the proton-pumping plasma-membrane ATPase Pma1 (Serrano, 1977; Serrano et al., 1986). Once inside the cell, maltose is hydrolysed by α-glucosidase (maltase) into two glucose molecules (Cook and Phillips, 1957). Maltotriose is assumed to be assimilated through the same system (Michaljanicova et al., 1982; Zastrow et al., 2001; Salema-Oom et al., 2005). In *S. cerevisiae*, the genes involved in maltose metabolism are clustered in *MAL* loci, which are located in the subtelomeric regions of multiple chromosomes. A *MAL* locus (“*MALx*”) includes three genes, encoding an α-glucoside transporter (*MALx1*), an α-glucoside hydrolase (maltase, *MALx2*) and a transcriptional regulator of gene cluster (*MALx3*). The number and identity of *MAL* loci is highly strain dependent (Goffeau et al., 1996; Nijkamp et al., 2012b; van den Broek et al., 2015), with five loci (*MAL1, MAL2, MAL3, MAL4*, and *MAL6*) having been identified and characterized in different strains (Cohen et al., 1985; Dubin et al., 1985; Charron and Michels, 1988; Charron et al., 1989; Chow et al., 1989; Michels et al., 1992). All *MALx1* genes share high sequence similarity (>95% at nucleotide level), except for *MAL11* (also referred to as *AGT1*), whose DNA sequence displays only 57% identity to the other four *MALx1* transporter genes (Han et al., 1995). In contrast to other *MALx1*-encoded transporters, Agt1 can efficiently transport α-glucosides other than maltose, such as trehalose (Plourde-Owobi et al., 1999), sucrose (Basso et al., 2011; Marques et al., 2017) and, importantly, maltotriose (Alves et al., 2008). The genome of S. *eubayanus* CBS12357 harbors four putative maltose transporter genes (*SeMALT1*; *SeMALT2, SeMALT3*, and *SeMALT4*) (Baker et al., 2015) which, however, have not yet been functionally analyzed. However, none of them appear to transport maltotriose since *S. eubayanus* CBS12357 is unable to grow on this trisaccharide (Hebly et al., 2015).

Maltose and maltotriose transport capacities in *S. pastorianus* cannot be described as a simple combination of transporters encoded by the genomes of current *S. cerevisiae* and *S. eubayanus* strains. While most *S. cerevisiae MAL* loci can be identified in *S. pastorianus* genomes, significant strain differences occur. For example, *AGT1* alleles in the *S. cerevisiae* subgenomes of the *S. pastorianus* strains Weihenstephan 34/70 (Nakao et al., 2009), CBS1483 (van den Broek et al., 2015) and A15 (Vidgren et al., 2005) carry a nucleotide insertion that interrupts their reading frames, causing a loss of function. A different, complete *AGT1* allele in these strains has been proposed to be derived from the *S. eubayanus* parental genome (Nakao et al., 2009; Vidgren and Londesborough, 2012), although no *AGT1* ortholog occurs in the genome of *S. eubayanus* CBS12357 (Baker et al., 2015). Additionally, *MTT1/MTY1* gene, which shows 90% identity with *MALX1*, has been identified in *S. pastorianus* strains and proposed to be involved in maltose and maltotriose transport at low temperature (Salema-Oom et al., 2005; Dietvorst et al., 2010; Cousseau et al., 2013). In total, maltose and maltotriose transport in *S. pastorianus* may involve up to ten different transporter genes which, taking into account the aneuploidy of its alloploid genome, could represent over 25 alleles in a single strain.

While most *S. pastorianus* strains rapidly ferment maltose, kinetics of maltotriose fermentation are typically much slower, resulting in extended fermentation process times and, in many cases, incomplete fermentation of this sugar (D'amore et al., 1989; Rautio and Londesborough, 2003; Rautio et al., 2007; Gibson et al., 2013). In addition to the genetic complexity of maltose and maltotriose metabolism in *S. pastorianus*, consumer acceptance issues preclude the use of targeted metabolic engineering approaches for improving sugar fermentation kinetics. Evolutionary engineering, also referred to as adaptive laboratory evolution (ALE) (Atwood et al., 1951), uses laboratory evolution to improve industrially relevant phenotypic characteristics and is a powerful non-GM approach for strain improvement (Vanee et al., 2012; Bachmann et al., 2015). Moreover, resequencing of the resulting evolved genomes can provide important insights into the genetic basis for the acquired improved performance (Oud et al., 2012).

The aim of the present study was to test an evolutionary engineering strategy for obtaining *S. pastorianus* strains with improved sugar fermentation kinetics and, in particular, with a faster conversion of maltotriose at the end of fermentation. To maintain a constant selective pressure for spontaneous mutants with an improved affinity for maltotriose, the model strain *S. pastorianus* CBS1483 (group II, Frohberg) was grown in carbon-limited chemostat cultures (Jansen et al., 2004) on a maltotriose-enriched sugar. After prolonged cultivation, single-cell lines were isolated and characterized in different culture systems, from bench-top fermenters up to 1,000 L pilot fermentation scale. Additionally, the performance of a selected strain was evaluated based on the quality of the bottled beer produced. Special attention was focused on the question of whether and to what extent laboratory evolution of an alloploid brewing strain for improved sugar fermentation kinetics affected profiles of flavor compounds in the final product.

## Material and methods

### Strains and maintenance

Yeast strains used in this study are listed in Table 1. Stock cultures were grown in YPD (10 g.L^−1^ Bacto yeast extract, 20 g.L^−1^ Bacto peptone and 20 g.L^−1^ glucose) until stationary phase, supplemented with sterile glycerol [final concentration 30% (v/v)] and stored at −80°C as 1 ml aliquots until further use.

**Table 1:** *Saccharomyces* strains used in this study.

**Strain name**	**Relevant genotype/description**	**References**
*S. cerevisiae* CEN.PK113-7D	*Mata SUC2 MAL2-8c*	Nijkamp et al., 2012a
*S. eubayanus* CBS12357	Isolated in Patagonia	(Libkind et al., 2011) CBS database^a^
*S. pastorianus* CBS1483	Group II brewer's yeast, Heineken's bottom yeast, July 1927	CBS database^a^
*S. pastorianus* IMS0493	Evolved CBS1483	This study
*S. pastorianus* IMS0495	Evolved CBS1483	This study
*S. pastorianus* IMS0507	Evolved CBS1483	This study
*S. pastorianus* IMS0508	Evolved CBS1483	This study

a *http://www.westerdijkinstitute.nl/collections/*.

### Media

Chemostat and bioreactor batch cultures were grown on synthetic medium (SM) containing 3.0 g.L^−1^ KH_2_PO_4_, 5.0 g.L^−1^ (NH_4_)_2_SO_4_, 0.5 g.L^−1^ MgSO_4_, 7 H_2_O, 1 mL.L^−1^ trace element solution, and 1 mL.L^−1^ vitamin solution (Verduyn et al., 1990). A sugar mixture (Dried Glucose syrup C plus 01987, Cargill, Haubourdin, France; sugar content (w/w): 2.5% glucose, 28.0% maltose, 42.0% maltotriose, 26.4% higher saccharides) was added to SM to a final concentration of fermentable sugars of 20 g.L^−1^ (SM-Mix). For cultivation on solid medium, SM or SM-Mix was supplemented with 2% (w/v) agar. SM used for anaerobic bioreactor cultivation was supplemented with ergosterol and Tween-80 (0.01 g.L^−1^ and 0.42 g.L^−1^, respectively; Verduyn et al., 1990) and with 0.15 g.L^−1^ of antifoam C (Sigma-Aldrich, Zwijndrecht, The Netherlands). In all cases, the pH was adjusted to 5.0 with 1 M HCl. For assays of maltose and maltotriose transport, yeast cells were pregrown in YPM (10 g.L^−1^ Bacto yeast extract, 20 g.L^−1^ Bacto peptone and 20 g.L^−1^ maltose).

For propagation and cultivation in E.B.C. (European Brewery Convention) tall-tubes (1977), strains were grown in aerated (18 ppm DO) 15° Plato industrial wort. Wort was produced from barley and wheat malt at the VTT Pilot Brewery (VTT Technical Research Centre of Finland Ltd, Espoo), and contained an extract of 15.0° Plato (62 g.L^−1^ maltose, 22 g.L^−1^ maltotriose, 16 g.L^−1^ glucose, and 4.6 g.L^−1^ fructose) and a free amino nitrogen (FAN) content of 269 mg.L^−1^ (Gibson et al., 2013). Pilot fermentation experiments were carried out in 1,300 L stainless steel tanks filled with 1,000 L of 15° Plato industrial wort. Prior to inoculation, the wort was aerated with 25 ppm of O_2_ and supplemented with zinc to a final concentration of 1.2 mg.L^−1^. The culture was pitched with 1.0 × 10^7^ cells.mL^−1^. Precultures were prepared by sequentially propagating cells from frozen stocks, into 50, 500 mL, 12, and 1,000 L pre-cultures on wort. All inoculum preparation stages were carried out at 20°C, except the last stage performed at 15°C.

### Culture conditions

#### Carbon-limited chemostat cultures

Laboratory evolution experiments were performed in four independent anaerobic chemostat cultures, grown on SM-mix at a dilution rate of 0.05 h^−1^. Two chemostat cultures (7L2 and 7R1) were grown in 2-L Applikon bioreactors (Applikon, Delft, The Netherlands), with a 1-L working volume of SM-Mix. The two other chemostat cultures (M1L and M2L) were performed in Multifors benchtop bioreactors (Infors, Velp, The Netherlands) with a working volume of 100 mL. Nitrogen gas (<10 ppm oxygen) was sparged through the cultures at 0.5 L.min^−1^ to ensure anaerobic conditions. Reactors were equipped with Norprene tubing (Saint-Gobain Performance Plastics, Courbevoie, France) and Viton O-rings (Eriks, Alkmaar, The Netherlands) to minimize oxygen diffusion. Culture pH was controlled at 5.0 by automated addition of 2.0 M KOH, the temperature was controlled at 16°C and stirrer speed was set at 800 rpm. Cultures were regularly sampled for analysis of OD_660_, biomass dry weight (only for 1-L working volume cultures) and metabolites (glucose, maltose, maltotriose, glycerol, and ethanol). Cultivation was continued until residual maltotriose concentrations stabilized. Chemostat cultures for comparing evolved isolates and the parental strain *S. pastorianus* CBS1483 were performed in 1-L working volume fermenters under the conditions described above. Cultures were analyzed when biomass concentrations in samples taken at 4, 6, and 9 volume changes after the onset of continuous cultivation varied by less than 2% (Jansen et al., 2004, 2005). Precultures were grown in 100-mL shake-flask at 20°C on SM-Mix medium and all chemostat cultures were inoculated at a cell density of 0.2 OD_660_U. L^−1^.

#### Bioreactor and tall-tube batch cultivation

Bioreactor batch cultures were grown in 2-L Applikon bioreactors (Applikon) with a 1-L working volume, equipped as described for chemostat cultivation, on SM-Mix. The cultures were grown at 15 or 20°C, pH was controlled at 5.0 and 100 mL inoculum cultures were grown at 20°C in 500 mL shake-flask on SM-Mix to an OD_660_ ranged between 10 and 12. The cultures were inoculated with 10 mL inoculum. Independent duplicate cultures were analyzed for each combination of strain and growth conditions.

For characterization of evolved isolates in wort, batch cultivation was performed at 15°C in 2-L cylindro-conical stainless-steel tall-tube fermentation vessels containing 1.9 L of wort. The wort medium was oxygenated to 18 mg L^−1^ before inoculation. Tall-tube experiments were preceded by a biomass propagation step on wort in the same set-up. When, at the end of this first fermentation, ethanol concentrations had remained constant for 24 h, yeast biomass was harvested by centrifugation and washed twice with sterile MilliQ-filtered water and resuspended to give a 20% (w/v) yeast suspension. The main fermentation was inoculated at a concentration of 5 g_wetbiomass_.L^−1^ (Gibson et al., 2013).

#### Pilot-scale fermentation experiments

The evolved strain *S. pastorianus* IMS0493 and parental strain CBS1483 were grown under industrial conditions. Independent duplicate fermentations were performed for both strains, in 1,300 L non-stirred stainless steel vessels containing 1,000 L of aerated wort (25 ppm O_2_) at a gravity of 15.0° Plato (Hough et al., 1971). Wort was supplemented with zinc to a final concentration of 1.2 mg.L^−1^. The main fermentation was inoculated at a cell density of 1.10^6^ cells.mL^−1^ and an initial temperature of 8°C, which was allowed to freely rise to 11°C during fermentation. When the gravity had decreased to 6.5° Plato, the temperature was increased to 14°C for a “ruh” phase (Hough et al., 1971). As diacetyl levels dropped below 20 ppb, the broth was cooled to 0°C within 14 h and lagered for 5 days. The beer was then filtered on a 0.4 μm membrane bottled in 30 cl capped bottles and pasteurized. Biomass from the final 1,000 L propagation step was harvested and stored in a cooled storage tank. Samples from pilot fermentations were taken at 24 h intervals. Bottled beers were also sampled and analyzed.

### Isolation of single cell lines from evolution experiments

Single cell lines were isolated from prolonged chemostat experiments by plating on solid SM-Mix and incubating plates anaerobically at 20°C. After three consecutive restreaks, one single-colony isolate from each evolution experiment was selected and characterized (Gonzalez-Ramos et al., 2016).

### Analytical methods

Optical density of cultures (OD_660_) was determined with a Libra S11 spectrophotometer (Biochrom, Cambridge, UK) at 660 nm. Biomass dry weight was determined by filtering duplicate 10 mL culture samples over preweighed nitrocellulose filters with a pore size of 0.45 μm. Filters were washed with 10 mL demineralized water, dried in a microwave oven (20 min at 350 W) and reweighed. Off gas of bioreactor fermentations was cooled to 4°C in a condenser and CO_2_ concentration was continuously monitored with an NGA 2000 analyser (Rosemount Analytical, Orrville, OH). For metabolite analysis, bioreactor culture samples were centrifuged and supernatants were analyzed with a Waters Alliance 2690 HPLC (Waters Co., Milford, MA) containing a Waters 2410 refractive-index detector, a Waters 2487 UV detector, and a Bio-Rad HPX-87H column (Bio-Rad, Hercules, CA) which was equilibrated and eluted with 0.5 g.L^−1^ H_2_SO_4_ at 60°C at a 0.6 mL.min^−1^ flow rate. Sugar concentrations in samples from tall tube fermentations were analyzed with a Waters HPLC with a Waters 2695 separation module and a Waters 2414 differential refractometer (Waters Co., Milford, MA) and an Aminex HPX-87H organic acid analysis column (Bio-Rad, Hercules, CA) equilibrated at 55°C and eluted with 5 mM H_2_SO_4_ in water at a 0.3 ml.min^−1^ flow rate.

Cell count and viability were determined using a NucleoCounter® YC-100™ (Chemometec A/S, Allerod, Denmark) according to the manufacturer's recommendations. The specific gravity, alcohol content and pH in pilot-scale experiments and bottled beer were analyzed after filtration using an Anton Paar density meter (Anton Paar GmbH, Graz, Austria). Concentration of fermentable sugars and volatile compounds (acetaldehyde, acetone, ethylformate, ethylacetate, methanol, ethylpropanoate, propanol, isobutanol, isoamylacetate, ethylcaproate, dimethyl sulfide, diacetyl, and 2,3-pentanedione) in these samples were determined by ultra-performance liquid chromatography (UPLC) (Waters Co) and gas chromatography, respectively.

To determine if two sets of data were significantly different from each other unpaired two-sample Student's *t*-test was used in Prism 4 (version 4.03) (Graphpad software Inc., La Jolla, CA).

### Analysis of sugar-uptake kinetics

Sugar-uptake kinetics by yeast cell suspensions were determined with [^14^C] labeled maltose and maltotriose [0.1 mCi.mL^−1^] (American Radiolabelled Chemicals, St Louis, MO). Prior to use ^14^C-labeled maltotriose was treated to remove impurities as described previously in Dietvorst et al. (2005). Transport rates were calculated from the radioactivity remaining inside the yeast cells after washing (Lucero et al., 1997). Yeast strains were pregrown in 100 mL YPM at room temperature and harvested at an OD_600_ between 3 and 7. After centrifugation, yeast pellets were washed with ice-cold water, followed by washing with ice-cold 0.1 M tartrate-Tris buffer (pH 4.2) and resuspended to a cell concentration of 200 mg yeast wet weight.mL^−1^ in the same buffer. Yeast suspensions were equilibrated to the 20°C assay temperature in a water bath. Experiments for maltose and maltotriose uptake rates were performed at different sugar concentration ranged from 1 to 25 mM. Sugar uptake was stopped after 60 s by addition of 5 mL ice-cold water and immediate filtration through a HVLP membrane (0.2 μm) (Millipore, Merck Life Science, Espoo, Finland). After rinsing the membrane with another 5 mL of ice-cold water, it was transferred to a scintillation cocktail (Optiphase Hisafe 3, PerkinElmer, Waltham, MA) and the radioactivity was counted in a scintillation counter (Tri-Carb 2810TR Low Activity Liquid Scintillation Analyzer, PerkinElmer). K_m_ and V_max_ were derived from Eadie-Hofstee plots fitting of the measured rates (Eisenthal and Cornish-Bowden, 1974). The statistical significance of observed differences between CBS1483 and IMS0493 strains was assessed by unpaired two-sample Student's *t*-test in Prism 4 (version 4.03) (Graphpad software Inc., La Jolla, CA).

### Genome sequencing

DNA was isolated from strains *S. pastorianus* IMS0493 and CBS1483 as previously described in van den Broek et al. (2015). Whole-genome sequencing was performed by Novogene (HK) Company Limited (Hong Kong, China). A DNA library was produced with the TruSeq DNA PCR-Free Library Preparation kit (Illumina, San Diego, CA). Paired-end libraries with 354 and 370-bp inserts were prepared for strains CBS1483 and IMS0493, respectively, and sequenced with an Illumina HiSeq2500 sequencer (Illumina). A total of 4.3 Gb and 4.6 Gb of 150-bp paired-end fragments were generated for IMS0493 and CBS1483 respectively. Chromosome copy numbers were estimated using the Magnolya software tool (Nijkamp et al., 2012a; Oud et al., 2013). For visualization purposes, assembled contigs and their copy number were mapped on an illumina reads based-assembly of *S. pastorianus* CBS1483 (ASM80546v1) (van den Broek et al., 2015). The copy number estimation of individual maltose transporter genes was performed by concatenating sequences of *SeMALT1* (GenBank Accession number: XM_018363333.1), *SeMALT2* (XM_018364778.1), *SeMALT3* (XM_018367005), *SeMALT4* (XM_018368187.1), *ScMAL11* (AJ012752.1), *ScMAL31* (NM_001178646.1), *ScMAL32* (NM_001178647.3), *SNF5*/YBR289W (Z36158.1), *MSH3*/YCR092C (M96250.1), *MES1*/YGR264C (Z73049.1), *SeBRN1*/SeYBL097W (XM_018363344.1) *SePDA1*/SeYER178W (XM_018364769.1) *SeATF1*/SeYOR377W (XM_), *SeGTR1*/SeYML121W (XM_018367018.1), *SePLC1*/SeYPL268W (XM_018368196.1) into an artificial contig onto 30.10^+6^ sequencing reads from IMS0493 or CBS1483 were mapped using Burrows Wheeler Aligner BWA (Li and Durbin, 2010) with default parameters. For each alignment 100 bp-average windows were calculated. For each gene 100-bp average windows data were collected and CBS1483 and IMS0493 data were statistically assessed for significant difference using unpaired two-sample Student's *t*-test in Prism 4 (version 4.03) (Graphpad software Inc., La Jolla, CA). Per gene average and standard deviation were calculated and plotted.

The raw sequencing data of strains IMSS0493, IMS0495 and IMS0508 are searchable at NCBI Entrez (http://www.ncbi.nlm.nih.gov/) under BioProject number PRJNA393253. The data of the parental strain CBS1483 can be found in BioProject PRJNA266750 (accession number SRP049726) (van den Broek et al., 2015).

## Results

### Suboptimal maltotriose fermentation by *S. pastorianus* CBS1483

In brewing, the term “attenuation” describes the extent to which brewing yeast completely converts wort extract into ethanol, CO_2_, yeast biomass and flavor compounds. Optimal attenuation requires efficient consumption of glucose, fructose, maltose as well as maltotriose and is a highly sought-after phenotypic trait in industrial *S. pastorianus* strains. Despite their history of domestication in wort, many lager yeasts exhibit incomplete or slow fermentation of the two major wort α-oligosaccharides, maltose and maltotriose.

**Figure 1:**
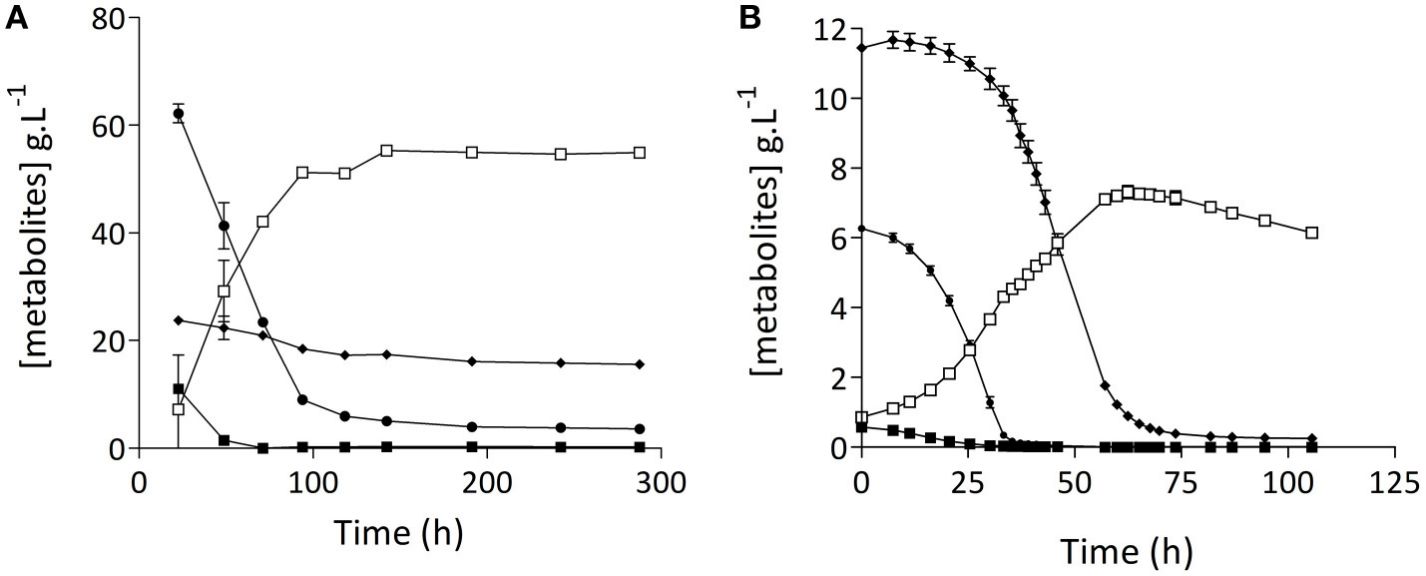
*Saccharomyces pastorianus* CBS1483 cannot complete wort α-oligosaccharide fermentation in anaerobic batch cultures: Metabolites determined (by HPLC) from filtered supernatant from *S. pastorianus* CBS1483 cultures. **(A)** Static fermentation in 2-L cylindrical fermentation tubes in 15° Plato wort at 15°C; **(B)** Stirred 2-L laboratory bioreactor cultures grown on synthetic medium (SM-Mix, containing 2.5% glucose, 28.0% maltose, 42.0% maltotriose, and 26.4% higher dextrin) at 16°C. (■) glucose, (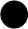) maltose, (♦) maltotriose, (□) ethanol. Graph shows average of biological independent duplicate fermentations ± standard error.

Since preliminary results indicated that the lager-brewing strain *S. pastorianus* CBS1483 could be an interesting model to study low attenuation due to suboptimal maltotriose fermentation kinetics in lager brewing strains, its growth and fermentation performance in 15° Plato wort was quantitatively analyzed in cylindrical 2-L stainless steel vessels at 15°C. Indeed, after 15 days of fermentation, residual concentrations of maltose and maltotriose remained at 3.2 ± 0.1 g.L^−1^ and 14.2 ± 0.1 g.L^−1^, respectively (Figure 1A). In these experiments, the fermentable sugars in wort were sequentially consumed. Glucose was fermented first, followed by maltose, while maltotriose consumption only began after 30 h. To investigate whether the incomplete utilization of maltose and maltotriose was caused by a limiting amount of available nitrogen in the wort, additional experiments with *S. pastorianus* CBS1483 were performed in stirred bioreactors on synthetic medium with excess nitrogen and lower overall sugar concentration (20 g.L^−1^ of a sugar mixture containing 2.5% glucose, 28.0% maltose, 42.0% maltotriose and 26.4% higher dextrins). As observed in the wort fermentations (Figure 1A), a sequential utilization of the fermentable sugars was observed (Figure 1B and Figure S1). Maltotriose consumption slowed down severely when its concentration reached ca. 1 g.L^−1^, and 0.25 g.L^−1^ maltotriose was left at the end of fermentation.

These data indicated that, irrespective of the initial sugar concentration and nitrogen source availability, *S. pastorianus* CBS1483 could not completely consume maltotriose. This phenotype was stronger under the higher gravity fermentation conditions representative of industrial brewing. It was therefore decided to use strain CBS1483 as a model to explore an evolutionary engineering strategy for improving the kinetics of maltotriose fermentation.

### Evolutionary engineering of *S. pastorianus* CBS1483 for improved growth substrate affinity

The batch cultivation experiments on wort and on maltotriose-enriched sugar mixtures (Figure 1) clearly indicated suboptimal kinetics of maltotriose fermentation in *S. pastorianus* CBS1483. In particular, a deceleration of fermentation at low maltotriose concentrations contributed to a poor attenuation in these mixed-substrate cultures. The ability of microorganisms to maintain high biomass-specific substrate conversion rates (q_s_) at growth-limiting concentrations of a substrate (C_s_), is referred to as the affinity for that substrate. When growth kinetics obey the Monod equation (q_s_ = q_s, max_ (C_s_/(C_s_ + K_s_))), affinity can be defined as q_s, max_/K_S_ (Button, 1985), in which q_s, max_ is the maximum biomass-substrate uptake rate and K_s_ is the substrate concentration at which q_s_ equals 50% of q_s, max_. Nutrient-limited chemostat cultivation confers a strong selective advantage to spontaneous mutants with an improved affinity for the growth limiting nutrient. During prolonged chemostat cultivation, a gradual “take over” of cultures by mutants with an improved affinity for the growth-limiting nutrient is often reflected by a progressive decrease of its residual concentration in the cultures (for examples see Jansen et al., 2004, 2005). To explore whether this concept can be applied to improve the affinity and fermentation kinetics of *S. pastorianus* CBS1483 for maltotriose, four independent chemostat experiments were performed. In these carbon-limited chemostat cultures, which were grown at a dilution rate of 0.05 h^−1^ and at 16°C, the carbon source consisted of a sugar mixture comprising 2.5% glucose, 28.0% maltose, 42.0% maltotriose, and 26.4% higher saccharides (w/w). Continuous cultivation was started when, in an initial batch cultivation stage on the same sugar mixture, maltotriose was the sole remaining carbon source in the bioreactor.

**Figure 2:**
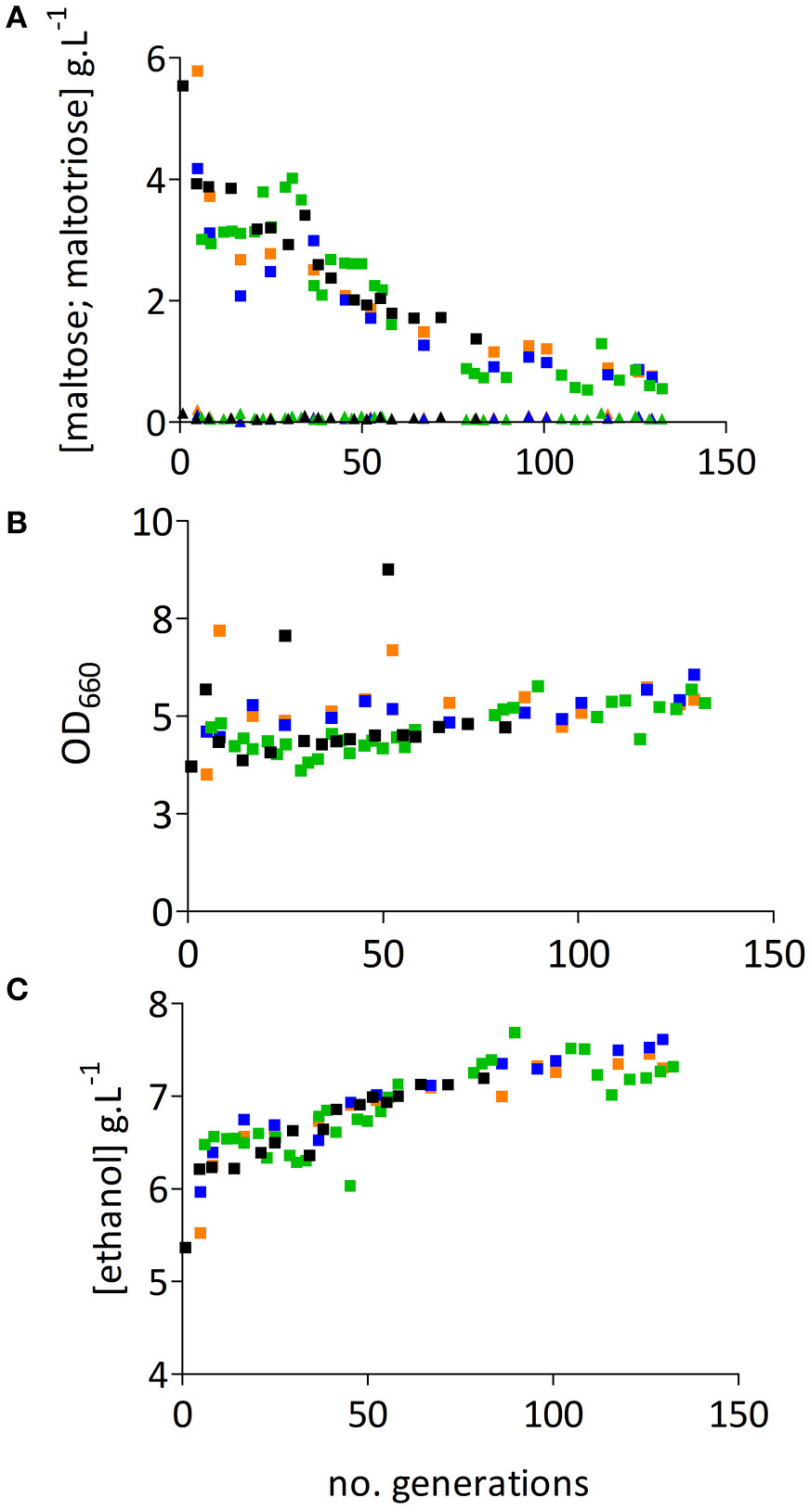
Evolutionary engineering of *S. pastorianus* CBS1483 in chemostat cultures. Graphs **(A–C)** display data from four independent evolution experiments in anaerobic, carbon- limited chemostat cultures, grown at 16°C on SM-Mix medium. Bioreactor working volumes were 100 mL in replicate evolution experiments M1L and M2L and 1 L in replicate experiments 7L2 and 7R1. **(A)** Residual extracellular maltose (▴) and maltotriose (■) concentrations. **(B)** OD measured at 660 nm (■). **(C)** Ethanol concentrations (■) during chemostat cultivation. Evolution experiment M1L is represented in blue, M2L in orange, 7R in green and 7L in black.

After 5 volume changes, concentrations of glucose and maltose in the four chemostat cultures were below detection level (<0.2 g.L^−1^ and <0.1 g.L^−1^, respectively), while maltotriose concentrations had not decreased below 3.0 g.L^−1^ (Figure 2A and Table S1). After approximately 30 generations, the residual maltotriose concentration in all four chemostat experiments gradually decreased until, after 80 generations, a reduction of about 70% was reached. In chemostat experiment 7R (Figure 2, green symbols) the maltotriose concentration decreased from 3.0 g.L^−1^ at the beginning of the culture to 0.56 g.L^−1^ after 132 generations (Figure 2A). The higher utilization of maltotriose at the end of this fermentation experiment was accompanied by increases of the biomass and the ethanol concentrations by 17% (Figure 2B) and 13% (Figure 2C) respectively.

### Single colony isolate IMS0493 expresses an improved phenotype representative of the final evolved culture

**Figure 3:**
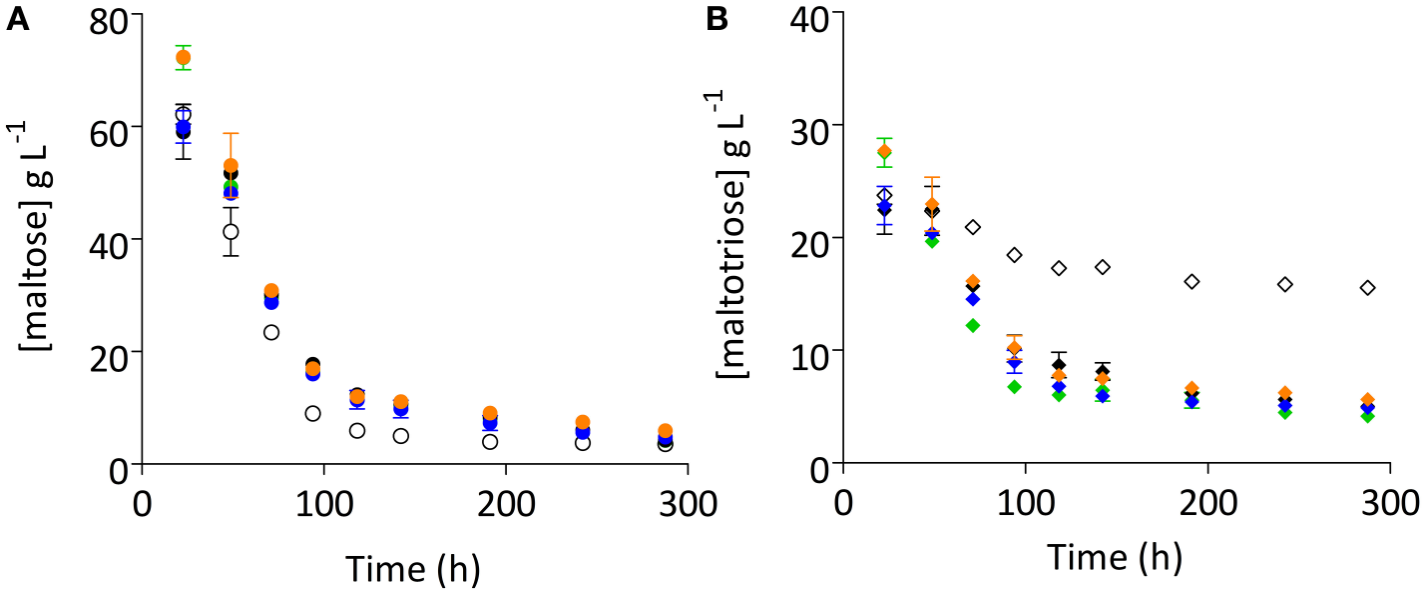
Improved maltotriose consumption of four independent single-colony isolates. Four single-colony isolates (IMS0508, IMS0507, IMS0493, and IMS0495) and their common parental strain *S. pastorianus* CBS1483 were grown in 2 L cylindrical fermentation tubes on 15° Plato wort at 15°C without mixing. Concentrations of the α-oligosaccharides maltose **(A)** and maltotriose **(B)** were measured by HPLC. The data presented show average ± mean deviation of independent duplicate fermentation experiments. Strain IMS0493 is shown in green, IMS0495 in black, IMS0507 in blue and IMS508 in orange. The parental strain CBS1483 is depicted with open circles and diamonds. Student's *t*-test analysis indicated significant improvement of maltotriose consumption in all four isolates (^IMS0493^*p*-value = 0.0011, ^IMS0495^*p*-value = 0.0132, ^IMS0507^*p*-value = 0.0057, ^IMS0508^*p*-value = 0.0438).

After 130 generations of selective chemostat cultivation on a sugar mixture, the fermentation performance of the evolved cultures could either reflect the characteristics of a mixed population or of a single, dominant enriched clonal population. To investigate whether pure cultures isolated from the evolution experiments exhibited an improved fermentation performance, single colonies were isolated from each evolution experiment. Four of these strains were grown on 15° Plato wort in cylindrical 2 L stainless-steel tall tubes, operated at 15°C. Single-colony isolates IMS0493, IMS0495, IMS0407, and IMS0508 clearly improved fermentation kinetics relative to their common parental strain CBS1483. In particular, residual maltotriose concentrations were significantly 2.8- to 3.7-fold lower (Student's *t*-test *p*-value = 0.0011) than observed for their common parental strain CBS1483 (Figures 1A, 3A, Figure S2). Although differences in residual maltose concentration were less pronounced and not statically significant (Student's *t*-test *p*-value = 0.5844), the evolved strains consistently showed a higher residual maltose concentration (18–66% increase relative to the parental strain). Of the four single-colony isolates, strain IMS0493 showed the best performance, with a 1.18-fold increase and a 3.7-fold decrease of the residual maltose and maltotriose concentrations, respectively, relative to *S. pastorianus* CBS1483 (Figures 1A, 3).

**Figure 4:**
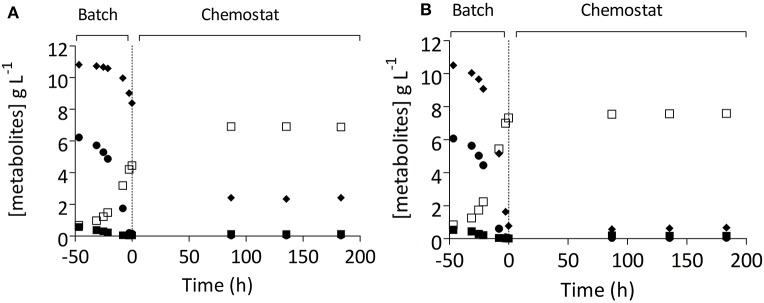
Residual sugars ((■) glucose, (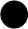) maltose, (♦) maltotriose), and ethanol (□) concentrations in carbon-limited chemostat cultures of *S. pastorianus* strains CBS1483 and IMS0493. The parental strain CBS1483 **(A)** and the evolved strain IMS0493 **(B)** were grown on SM-Mix (2.5% glucose, 28.0% maltose, 42.0% maltotriose, 26.4% higher dextrin) at 16°C in 1-L working volume anaerobic chemostat cultures at a dilution rate of 0.05 h^−1^. The vertical line depicts transition from the batch phase (−50 to 0 h) to the continuous-cultivation phase (from 0 h onwards). Sugar concentrations in an independent duplicate experiment differed by less than 2.5%.

To test whether strain IMS0493 phenotypically resembled the evolving population from which it had been isolated, its growth and metabolite profile were compared with those of its parental strain CBS1483 in carbon-limited mixed-sugar (SM-Mix) chemostat cultures grown at a dilution rate of 0.05 h^−1^ (Figure 4, Table 2). Chemostat cultures of strain IMS0493 rapidly reached a steady state in which extracellular metabolite profiles resembled those observed at the end of the evolution experiment, whilst strain CBS1483 showed the same high residual maltotriose concentrations that were observed at the beginning of the evolution experiments. These results confirmed that, during chemostat-based evolution, strain IMS0493 had acquired an improved affinity for maltotriose which contributed to lower residual sugar concentrations and higher biomass concentrations in steady-state cultures (Table 2) and better attenuation in batch cultures on wort (Figure 3). A faster and more complete conversion of maltotriose by strain IMS0493, relative to it parental strain CSB1483, was also observed in controlled bioreactor batch cultures, grown at 16°C on SM-Mix (Figure 3). In these cultures, CO_2_ production profiles of the non-evolved strain CBS1483 exhibited two distinct peaks, reflecting diauxic utilization of first maltose and then maltotriose (Figure S1). In contrast, the CO_2_ profile of the evolved isolate IMS0493 showed a single peak (Figure S1) which, as confirmed by analysis of sugar concentrations, reflected co-utilization of glucose, maltose and maltotriose (Figure 3 and Figure S1).

**Table 2:** Evolved single colony isolate IMS0493 shows improved maltotriose fermentation kinetics compared to its parental strain *S. pastorianus* CBS1483 in a chemostat culture.

	**CBS1483**	**IMS0493**
dry weight g.L^−1^	1.23	1.37
Y_x/s_ g.g^−1^	0.08	0.09
q_maltotriose_ mmol.g^−1^ h.h^−1^	−0.70	−0.76
q_maltose_ mmol.g^−1^ h.h^−1^	−0.74	−0.66
q_ethanol_ mmol.g^−1^ h.h^−1^	5.98	6.00
C-recovery (%)	97.87	96.37
Residual [maltotriose] g.L^−1^	2.41	0.68
Residual [maltose] g.L^−1^	0.04	0.06
Final [ethanol] g.L^−1^	6.88	7.60
K_s_ maltose mM	3.6	3.9
K_s_ maltotriose mM	7.2	6.5

*Biomass yield and rates were determined from steady state measurements of oligosaccharide-limited chemostat cultivations and K_S_ were calculated from the ^14^C labeled maltose and maltotriose uptake assays*.

### Improved maltotriose transport capacity

**Figure 5:**
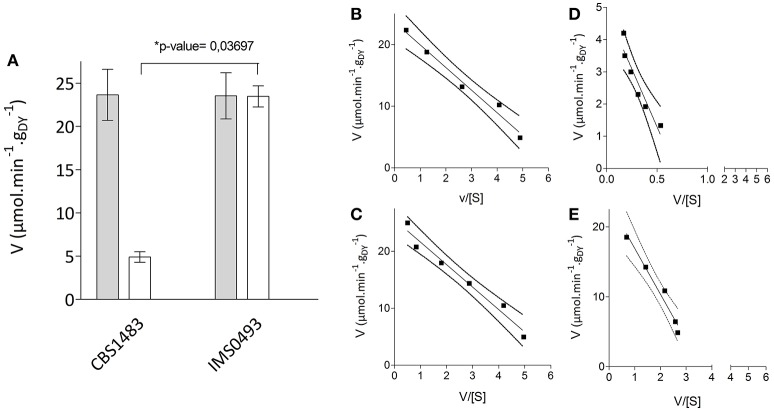
Uptake kinetics of labeled oligo-saccharides of *S. pastorianus* strains CBS1483 and IMS0493. **(A)** Maximum uptake rates of labeled [^14^C] maltose (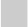) and labeled [^14^C] maltotriose (□) of *S. pastorianus* strains CBS1483 and IMS0493 measured at 20°C derived from Eadie-Hofstee fits. Values presented are averages ± mean deviation of biological duplicates. ^*^Indicates the significant differences between the tested strains with the corresponding Student's *t*-test *p*-value. **(B)** Determination of K_m_ and V_max_ for maltose of *S. pastorianus* CBS1483 from Eadie-Hofstee plots (Eisenthal and Cornish-Bowden, 1974) with maltose concentration ranged from 2.5 to 25 mM. Intercept with the Y-axis defined V_max_ (23.7 μmol.min^−1^.g_DY_^−1^) and the slope defined –Km (−3.6 mM). **(C)** Determination of K_m_ and V_max_ for maltose of *S. pastorianus* IMS0493 from Eadie-Hofstee plots with maltose concentration ranged from 2 to 25 mM. Intercept with the Y-axis defined Vmax (23.5 μmol.min^−1^.g_DY_^−1^) and the slope defined –Km (−3.9 mM). **(D)** Determination of K_m_ and V_max_ for maltotriose of *S. pastorianus* CBS1483 from Eadie-Hofstee plots with maltotriose concentration ranged from 1 to 25 mM. Intercept with the Y-axis defined Vmax (4.9 μmol.min^−1^.g_DY_^−1^) and the slope defined –Km (−7.2 mM). **(E)** Determination of K_m_ and V_max_ for maltotriose of *S. pastorianus* IMS493 from Eadie-Hofstee plots with maltotriose concentration ranged from 2.5 to 25 mM. Intercept with the Y-axis defined Vmax (23.5 μmol.min^−1^.g_DY_^−1^) and the slope defined –Km (−6.5 mM). The dash lines represent the 95% confidence interval of the regression line.

To investigate the role of maltotriose transport kinetics in the improved affinity of the evolved strain IMS0493 for this trisaccharide, sugar-uptake assays were performed at several [^14^C-] labeled maltose or maltotriose concentrations. An Eadie-Hofstee fit of the sugar rates revealed that the maximum maltose-uptake rates of the strain IMS0493 and CBS1483 were similar (Figure 5). Substrate-saturation constants (K_m_) of strains IMS0493 and CBS1483 were also very similar for maltose (3.9 and 3.6 mM, respectively) but also for maltotriose (6.5 and 7.2 mM, respectively). In contrast, the V_max_ for maltotriose uptake of the evolved strain IMS0493 was over 4-fold higher (Student's *t*-test *p*-value = 0.03697) than that of the parental strain CBS1483 [23.5 and 4.9 μmol.min^−1^.(g_dry biomass_)^−1^ respectively; Figure 5]. The transport capacity for maltose of both strains was approximately 23 μmol.min^−1^.(g_dry biomass_)^−1^ (Figure 5). These results are consistent with an evolutionary adaptation to sugar-limited chemostat cultivation in which a higher affinity (q_s, max_/K_m_) for maltotriose primarily resulted from increased expression and/or an improved transport capacity (mol_maltotriose_.(mol _transporter_)^−1^.s^−1^) of one or more maltotriose transporters in the yeast plasma membrane.

### Whole-genome resequencing of evolved strain

**Figure 6:**
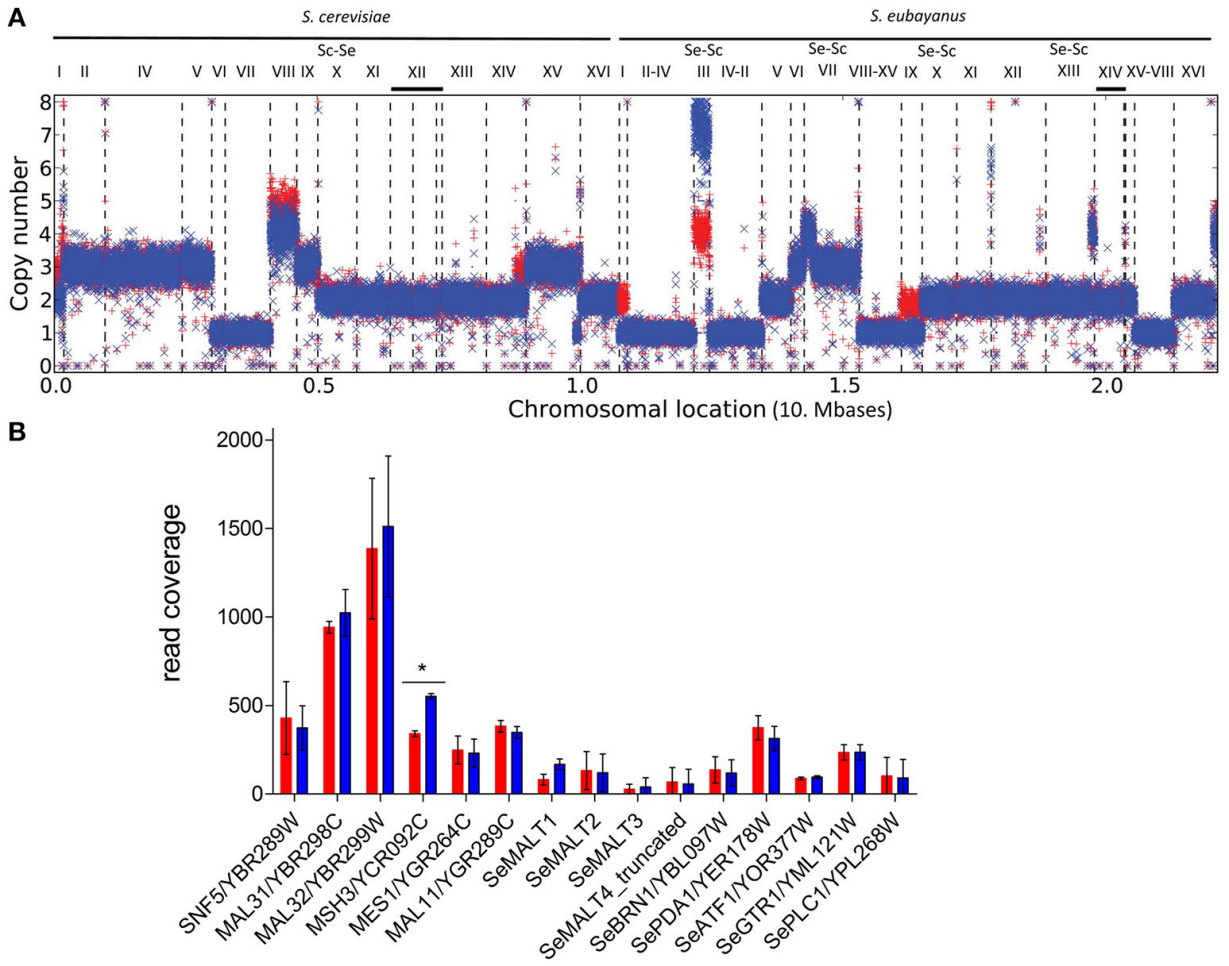
Ploidy differences between the evolved mutant IMS0493 (blue) and the ancestral parent *S. pastorianus* CBS1483 (red). **(A)** The graph represents the ploidy prediction generated with the Magnolya algorithm (Nijkamp et al., 2012b). Contigs that were de novo assembled by Newbler (454 Life Sciences) and aligned to the reference *S. pastorianus* CBS1483 genome sequence (ASM80546v1) (van den Broek et al., 2015) using NUCMER (MUMmer, version 3.21; www.mummer.sourceforge. net). **(B)** CBS1483 (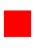) and IMS0493 (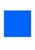) sequencing read mapping on an artificial contig resulting of the concatenation of *S. cerevisiae MAL11, MAL31, MAL32, SNF5, MSH3, MES1* and *S. eubayanus MALT1, MALT2, MALT3, MALT4*^*truncated*^*, SeBRN1, SePDA1, SeATF1, SeGTR1, SePLC1* using Burrows-Wheeler Aligner BWA (Li and Durbin, 2010). Coverage was calculated over 100 bp-coverage windows. Per gene difference significance of set of 100 bp coverage windows from CBS1483 and IMS0493 was statistically assessed using Student's *t*-test (*p*-value < 0.05). The data represented are averages and standard deviations of per gene 100 bp coverage windows. ^*^Indicates a difference in coverage between CBS1483 and IMS0493 deemed significant using Student's *t*-test (*p*-value < 0.05).

The alloploid genomes of *S. pastorianus* strains, including the reference strain CBS1483 used in the present study (van den Broek et al., 2015), complicate identification of point mutations by short-read resequencing technologies. However, chromosome copy number analysis (Nijkamp et al., 2012a) indicated that, in the evolved strain IMS0493, a 2-fold amplification had occurred of a large part of CHRIII, while copy numbers of chromosomes ScI, ScVIII, SeI, SeIX and a segment of the right arm of ScXIV had decreased. The copy number of the affected part of CHRIII had increased from four copies in strain CBS1483 to eight copies in strain IMS0493 (Figure 6A). *S. pastorianus* CBS1483 carries only one version of this chromosome, which is composed of the left arm, centromere and a large part of the right arm of *S. eubayanus* CHRIII and the end of the right arm of *S. cerevisiae* CHRIII (van den Broek et al., 2015). The *MAL2* locus, which is located on the subtelomeric region of the *S. cerevisiae* right arm of CHRIII, seemed not to be affected by the amplification. However, the copy number estimation of individual maltose transporter encoding gene was disrupted by the inability to accurately assemble paralogous genes (e.g., *SeMALT2* and *SeMALT4* or *MAL31, MAL21, MAL41* and *MAL61*) with short sequencing reads. Thus, an attempt to get quantitative estimation of maltose transporter genes was performed by mapping sequencing reads from CBS1483 and IMS0493 on an artificial contig composed of the sequences of all four *S. eubayanus* maltose transporter genes (*SeMALT1, SeMALT2, SeMALT3* and the truncated *SeMALT4*, Baker et al., 2015), two *S. cerevisiae MALx1* genes (*MAL11, MAL31*,). To standardize the mapping results, control genes (*SNF5*/YBR289W, *MSH3*/YCR092C, *MES1*/YGR264C, *SeBRN1*/SeYBL097W, *SePDA1*/SeYER178W, *SeATF1*/SeYOR377W, *SeGTR1*/SeYML121W, *SePLC1*/SeYPL268W) located on chromosomes harboring a *MAL* gene and having no paralogs were selected and together with the highly conserved maltase gene *MAL32* present in all *S. cerevisiae MAL* loci added to the artificial contig. The sequence reads mapping from IMS0493 and its ancestor CBS1483 on these concatenated sequences corroborated the amplification of a large fraction of CHRIII as coverage of *MSH3* was deemed statistically different and the average 1.8-fold higher supporting a doubling of ScCHRIII. All other tested markers did not reveal any significant difference, suggesting that maltose transporter encoding genes were neither gained nor lost in the evolved strain IMS0493 (Figure 6B and Figure S3).

### Technology transfer: fermentation performance at pilot scale

**Figure 7:**
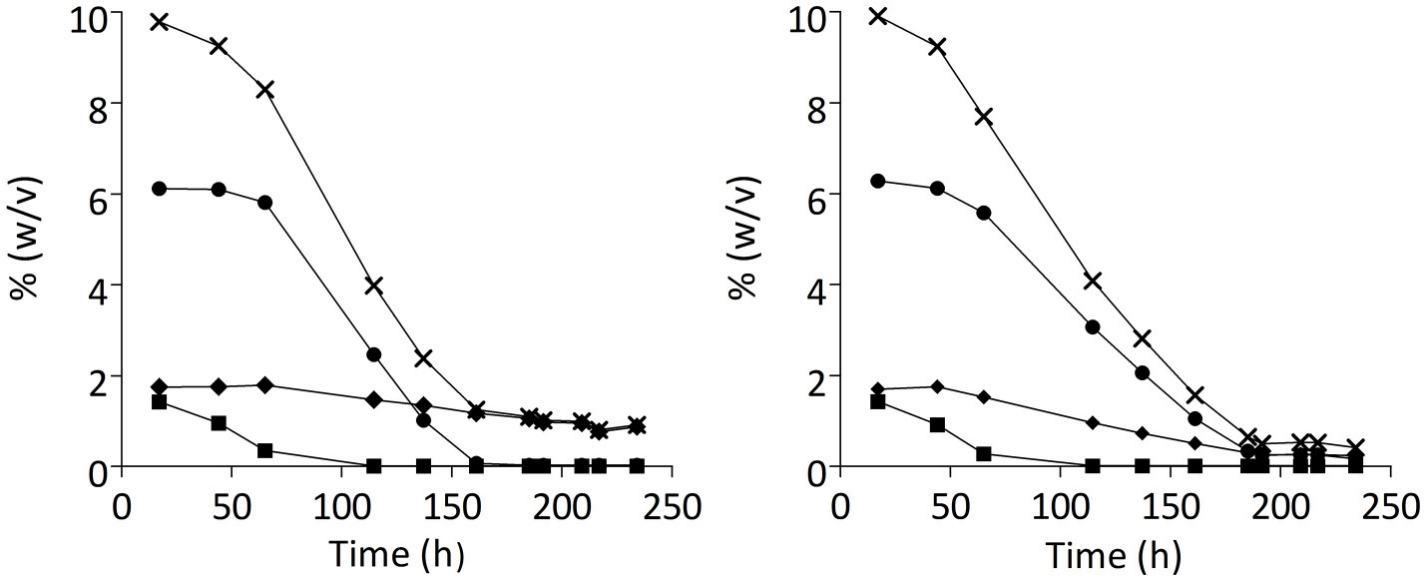
Sugar consumption profiles of *S. pastorianus* strains CBS1483 **(A)** and IMS0493 **(B)** in high- gravity wort at 1,000-L pilot scale. Total extract (X) and Extracellular metabolites [(■) glucose, (●) maltose, (♦) maltotriose] concentrations (expressed % weight/volume) in fermented wort samples were determined by liquid chromatography. Sugar concentrations in an independent duplicate experiment differed by less than 2.5%.

Laboratory evolution is a powerful tool to develop strains with improved, innovative traits. Although often performed in academic studies (Novick and Szilard, 1950; Ferea et al., 1999; Wisselink et al., 2009; Gonzalez-Ramos et al., 2013; Oud et al., 2013), these are rarely accompanied by assessment at pilot or full industrial scale. To investigate the industrial relevance of the evolutionary engineering strategy described above, the evolved strain IMS0493 and its parental strain CBS1483 were each tested in duplicate industrial pilot-scale (1,000 L) beer-fermentation experiments on a high-gravity 15° Plato wort (Figure 7). These experiments showed that the acquired phenotypes of the evolved strain were also expressed under industrial conditions. In particular, IMS0493 fermentation showed a significantly lower residual concentration of total fermentable sugars at the end of fermentation (Student's *t*-test *p*-value = 0.021944) (Table 3). Total fermentable sugars left in the green beer fermented by IMS0493 were 53% lower than in reference fermentations with its non-evolved parent strain (Table 3). This improvement was predominantly caused by a more complete conversion of maltotriose, whose residual concentration was reduced by 72% in comparison to residual concentration measured in beer fermented by CBS1483 (Table 3). Although residual maltose concentrations remained below 2 g.L^−1^, they were 83% higher in experiments with the evolved strain.

**Table 3:** Analysis of green and standardized beers fermented with the evolved isolate IMS0493 and the parental reference CBS1483 in 1,000-L scale.

	**Unit**	**CBS1483**	**IMS0493**	
		**Average. ± mean dev**.	**Average. ± mean dev**.	***p*-value**
**GREEN BEER**				
Ethanol by volume	%(V/V)	6.32 ± 0.01	6.74 ± 0.03	0.04333^*^
Total higher alcohols	mg.L^−1^	105.6 ± 0.71	121.9 ± 0.78	0.04107^*^
Diacetyl (2,3-butanedione)	mg.L^−1^	29.6 ± 8.08	18.9 ± 2.40	0.508841
Total fermentable sugars left	%(m/V)	0.91 ± 0.05	0.47 ± 0.03	0.02194^*^
**STANDARDIZED BEER**				
Ethanol by volume	%(v/v)	5.00	5.00	
pH		4.26 ± 0.01	4.29 ± 0.01	0.062661
**HIGHER ALCOHOLS**				
Propanol	mg.L^−1^	9.58 ± 0.36	10.84 ± 0.26	0.079166
Isobutanol	mg.L^−1^	12.33 ± 0.30	14.70 ± 0.02	0.01552^*^
Amyl alcohol	mg.L^−1^	61.04 ± 1.43	64.92 ± 0.50	0.081175
Total higher alcohols	mg.L^−1^	82.95 ± 1.37	90.46 ± 0.74	0.04107^*^
**ESTERS**				
Isoamylacetate	mg.L^−1^	3.11 ± 0.14	3.26 ± 0.07	0.425926
Ethylacetate	mg.L^−1^	23.25 ± 0.67	23.24 ± 0.66	0.581175
**VICINAL DIKETONES**				
2.3-Butanedione (Diacetyl)	μg.L^−1^	23.21 ± 7.54	14.03 ± 1.31	0.498791
2.3-Pentanedione	μg.L^−1^	21.96 ± 8.63	17.71 ± 1.66	0.802657
**OTHERS**				
Acetaldehyde	mg.L^−1^	2.99 ± 0.43	5.31 ± 0.35	0.175915
Dimethyl sulfide (DMS)	μg.L^−1^	31.42 ± 2.10	48.03 ± 3.15	0.156564
**SUGARS**				
Glucose	%(w/v)	Bdl^@^	Bdl^@^	NA^#^
Fructose	%(w/v)	0.02 ± 0.00	0.02 ± 0.00	NA^#^
Maltose	%(w/v)	Bdl^@^	0.12 ± 0.02	0.052634
Maltotriose	%(w/v)	0.69 ± 0.02	0.20 ± 0.00	0.04237^*^
Total fermentable sugars	%(w/v)	0.71 ± 0.02	0.35 ± 0.02	0.0219^*^

*Higher alcohols, esters and vicinal diketones were determined by gas chromatography and sugars and ethanol were measured by liquid chromatography. Extract data were determined using an alcolyzer beer analyzing system (Anton-Paar). Values presented are averages ± mean deviation of biological duplicates. The p-values were calculated using Student's t-test*.

* *Indicates a difference between CBS1483 and IMS0493 deemed significant using Student's t-test (p-value < 0.05)*.

@ Bdl, below detection limit;

# *NA, Not Applicable*.

To explore whether the improved maltotriose fermentation kinetics of evolved strain IMS0493 affected other brewing-related strain characteristics, beers from the pilot fermentations were analyzed. Consistent with the analysis on the 1,000-L fermentation experiments, the ethanol concentration in beer brewed with strain IMS0493 was 6% higher than in beer made with the reference strain, while the concentration of residual fermentable sugar was 48% lower (Student's *t*-test *p*-value = 0.043339 and 0.021944 respectively) (Table 3). These results confirmed that the increased fermentation efficiency seen during laboratory experiments could be transferred to an industrial setup (Huuskonen et al., 2010). This increase in ethanol concentration would account for a gain of 7.4% in volumetric productivity if the green beer were standardized to 5% (v/v) ethanol (Table 3). To investigate whether such standardized beers would comply with lager beer quality standards, aroma compounds were measured (Table 3). Total higher alcohols concentration in standardized bottled beer made with the evolved strain were 9% higher than in similar beer brewed with *S. pastorianus* CBS1483 but remained within specifications. Additionally, a 40% lower diacetyl concentration was observed in bottled beer produced with the evolved strain IMS0493 (Table 3). The level measured in IMS0493 (14 μg.L^−1^) was far below the sensory threshold of 20 μg.L^−1^. In comparison the level measured in the non-evolved CBS1483 were just above the threshold (23 μg.L^−1^). Altogether, the aroma profile of beers produced with the two strains met common quality standards for lager beers.

## Discussion

In chemistry and fuel applications, yeast strain development has been intensified and accelerated by novel genome-editing tools such as CRISPR-Cas9 (Doudna and Charpentier, 2014). These techniques enable highly accurate and, when desired, simultaneous gene deletion, nucleotide editing and chromosomal integration of novel genes (DiCarlo et al., 2013; Mans et al., 2015; Jakociunas et al., 2016). Although regulatory frameworks do not preclude use of genome editing for brewer's yeast strain improvement, consumer concerns about use of genetically modified (GM) organisms for food and beverage production discourage industry from implementing this option (Varzakas et al., 2007). In contrast, classical, non-targeted mutagenesis by irradiation (UV, X-ray) or chemical compounds (e.g., ethyl methanesulfonate) continue to be successfully applied in brewer's yeast (Blieck et al., 2007; Huuskonen et al., 2010) but requires intensive screening for mutants with beneficial mutations. Additionally, mutagenesis increases the likelihood of secondary mutations, which may negatively affect industrially relevant traits that are difficult to screen in high-throughput set-ups, such as the complex balance of flavor and aroma compounds in the final product.

Evolutionary engineering, is a non-GM technique that is highly suitable for strain improvement in food biotechnology (Bachmann et al., 2015). When a relevant aspect of strain performance can be experimentally linked to growth rate or survival, better performing strains can often be obtained within 50–200 generations, without requiring active mutagenesis (Cadiere et al., 2011; Ekberg et al., 2013; Gonzalez-Ramos et al., 2013, 2016; Tilloy et al., 2015). In the present study, an *S. pastorianus* strain evolved in laboratory chemostat cultures showed a strongly improved affinity for maltotriose. While the evolutionary strategy explored in this study was designed to improve affinity for maltotriose, costs of pure maltotriose compelled us to use a mixed-sugar substrate, which also contained substantial amounts of maltose. This choice likely contributed to the acquisition of a maltose/maltotriose co-consumption phenotype acquired by the evolved *S. pastorianus* strain IMS0493, which enabled a faster wort fermentation (Figures 3, 7, Figure S2). Accelerated fermentation probably contributed to a faster reduction of diacetyl and, thereby, to the observed lower concentrations of this off-flavor in beer brewed with the evolved strain. The aneuploid, alloploid genomes of lager brewing yeasts are diverse and dynamic with respect to chromosomal copy number variations (Gorter de Vries et al., 2017). Indeed, whole-genome sequencing revealed several copy number variations in strain IMS0493, which evolved for improved maltotriose affinity. The genome dynamics of industrial lager brewing yeast might also be associated with decreased diacetyl formation in IMS0493. Acetolactate is converted to diacetyl under the action of acetolactate synthase, an enzyme comprising two subunits. The catalytic subunit is encoded by *ILV2* and the regulatory subunit encoded by *ILV6* a gene located on CHRIII. Although the *ILV6* copy number has been associated with variation in diacetyl production in *S. pastorianus*, a reduction in copy number was associated with lower diacetyl (Duong et al., 2011) while overexpression of either of the *ILV6* alleles led to increased diacetyl formation (Gibson et al., 2015). These results would disagree with the results of this study which would associate a doubling of *SeILV6* copy number with a reduction of diacetyl. This would suggest that other genetic elements might influence the level of this undesired metabolite. Extensive chromosome copy number variations observed in IMS0493 did not negatively affect flavor profile in pilot-scale fermentations. The properties acquired by the evolved strain enabled an increased production of beer from the same amount of wort, with a reduced maltotriose content and a flavor profile that was fully compatible with lager brewing specifications. This result demonstrates the potential of evolutionary engineering to improve specific traits of brewing yeasts without disturbing other industrially relevant phenotypes.

Sugar-transport studies indicated that the improved affinity of evolved strain IMS0493 was caused by a 3-fold higher maltotriose transport capacity than that of the parental strain. However the genetics of maltose and maltotriose transport in *S. pastorianus* have not yet been fully resolved by functional analysis of individual oligosaccharide transporters. This is especially relevant for a novel *SeAGT1* allele identified in *S. pastorianus* (Nakao et al., 2009; Vidgren and Londesborough, 2012; Cousseau et al., 2013) but that so far could not be identified in available *S. eubayanus* sequences (Baker et al., 2015; Hebly et al., 2015).

Identification of causative mutations remains the next challenge. Whole-genome sequencing, using Illumina short-read technology, which allows for accurate analysis of chromosomal copy number variations (Nijkamp et al., 2012a), did not reveal changes in the copy number of known or putative sugar transporter genes in the evolved strain *S. pastorianus* IMS0493. The genetic basis for improved maltotriose uptake in this strain is therefore more complicated than a simple amplification of a known transporter gene. In haploid or homozygous yeast genomes, single-nucleotide mutations in non-repetitive sequences can be easily identified by short-read sequencing technologies (Gonzalez-Ramos et al., 2013; Caspeta et al., 2014; Caspeta and Nielsen, 2015). However, in alloploid brewing yeast genomes, their identification remains a critical challenge, despite fast developments in sequencing technology (Oud et al., 2012). While tools such as GATK (McKenna et al., 2010) selectively search for heterozygous positions in diploid genomes, no such tools are currently available to identify heterozygous single-nucleotide mutations in aneuploid genomes. In the case of *S. pastorianus*, such an identification is especially challenging because the *S. cerevisiae* and *S. eubayanus* subgenomes are closely related and, locally, share near-complete sequence identity (Libkind et al., 2011; Baker et al., 2015; Hebly et al., 2015). Furthermore, recent studies have established that *S. pastorianus* strains can be highly aneuploid, with individual chromosome copy numbers ranging from one to five in a single genome (Nakao et al., 2009; Hewitt et al., 2014; Walther et al., 2014; van den Broek et al., 2015; Okuno et al., 2016). Consequently, phasing of two or more variable positions in a single gene is only feasible if they are captured in a single sequencing read. Similar to the problems encountered in this study, genome-wide expression analysis could not identify the exact molecular basis for improved performance of mutagenized *S. pastorianus* selected for growth in very high gravity wort (Blieck et al., 2007) and at high osmolarity (Ekberg et al., 2013). Research in this area is likely to profit from fast developments in long-read technology (e.g., Pacific Biosystems and Oxford Nanopore), which will facilitate reconstruction of single alleles and haplotype chromosome versions and, thereby, development of new bioinformatics solutions to identify relevant mutants in hybrid, highly aneuploid genomes.

## Author contributions

AB, JP, NK, JG, BG, and JD designed experiments. AB, JP, NK, JG, Mv, FM, BG, and JD critically analyzed the results. AB and NK performed the experiments. AB, JP, and JD wrote the manuscript. All authors read and approved the final manuscript.

### Conflict of interest statement

The authors declare that the research was conducted in the absence of any commercial or financial relationships that could be construed as a potential conflict of interest. It worth mentioning that JG and NK are employed by HEINEKEN Supply Chain, Global Innovation & Research (Zoeterwoude, NL).
