# Supplementary material for: Evolutionary Engineering in Chemostat Cultures for Improved Maltotriose Fermentation Kinetics in Saccharomyces pastorianus Lager Brewing Yeast
Source: Front Microbiol. 2017 Sep 8;8:1690. doi: 10.3389/fmicb.2017.01690 (PMC5596070; doi:10.3389/fmicb.2017.01690)
Supplement: Supplementary file 1 [file Presentation1.pdf]

Supplementary data/ Table/ Figures

Table S1: Independent evolution lines showed comparable results concerning residual metabolite concentration. Residual maltotriose concentrations for evolution culture at time points (generations) of isolation of single colonies.

**Fermenter no.**	**7L (2)**	**7R**	**M1L**	**M2L**
**Culture volume (mL)**	1000	1000	100	100
**Age (generations)**	81	132	125	125
**Optical density (OD_{660})**	4.7	5.34	5.4	5.3
**Residual maltotriose (g L^{-1})**	1.38	0.55	0.87	0.83
**Single isolate derived**	IMS0495	IMS0493	IMS0507	IMS0508

Legends of supplemental figures

Figure S1: *S. pastorianus* CBS1483 displays a sequential fermentation of glucose, maltose, and maltotriose. A Metabolites (■) glucose, (●) maltose, (♦) maltotriose and (☐) ethanol determined (by HPLC) from filtered samples taking during cultivation of *S. pastorianus* CBS1483 in SM-Mix at 16 ^{o}C. B Percentage CO_2 determined by off gas analysis during cultivation of *S. pastorianus* strain CBS1483 on SM-Mix at 16 ^{o}C. C Metabolites (■) glucose, (●) maltose, (♦) maltotriose and (☐) ethanol determined (by HPLC) from filtered samples taking during cultivation of *S. pastorianus* IMS0493 in SM-Mix at 16 ^{o}C. D Percentage CO_2 determined by off gas analysis during cultivation of *S. pastorianus* strain IMS0493 on SM-Mix at 16 ^{o}C.

Figure S2: Single colony isolates of four independent evolution lines show highly improved maltotriose fermentation kinetics compared to the initial *S. pastorianus* strain CBS1483 but decreased maltose fermentation. Metabolites (■) glucose, (●) maltose, (♦) maltotriose and (☐) ethanol determined (by HPLC) from filtered samples taking during cultivation of **A** *S. pastorianus* strain (duplication of the data from figure 1A) CBS1483 and four evolved isolates (**B** = IMS0493, **C** = IMS0495, **D** = IMS0507, **E** = IMS0508) on 15 ^{o} Plato wort at 15 ^{o}C in static fermentation in 2L

cylindrical fermentation tubes. Graph shows average of two independent duplicate fermentations including error bars.

Figure S3: Ploidy differences between the evolved mutant IMS0493 (blue) and the ancestral parent *S. pastorianus* CBS1483 (red) over SeCHRII-IV, SeCHRV, SeCHRXIII, SeCHRXVI, ScCHRII, ScCHRVII, ScCHRVIII, ScCHRXIand SeCHRXIII-ScCHRXIII. A- The graph represents the ploidy prediction generated with the Magnolya algorithm (Nijkamp et al., 2012b). Contigs that were de novo assembled by Newbler (454 Life Sciences) and aligned to the reference *S. pastorianus* CBS1483 genome sequence (ASM80546v1) (van den Broek et al., 2015) using NUCMER (MUMmer, version 3.21; (www.mummer.sourceforge.net). The arrows indicate the position of MAL loci in S. pastorianus parental genomes of S. cervisiae and S. eubayanus.

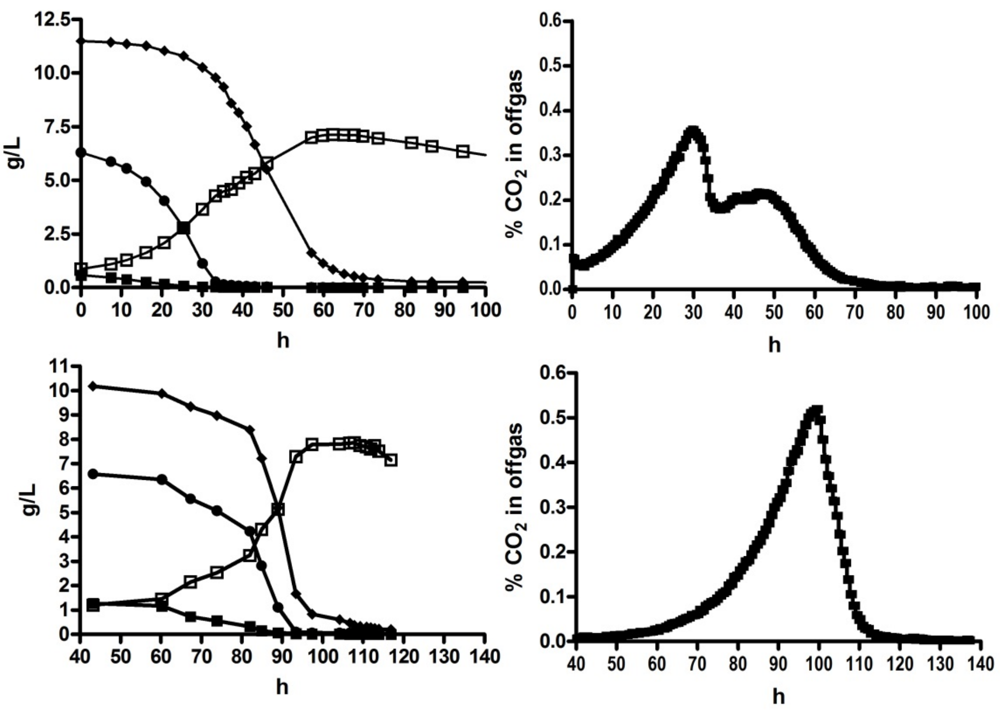

Figure S1

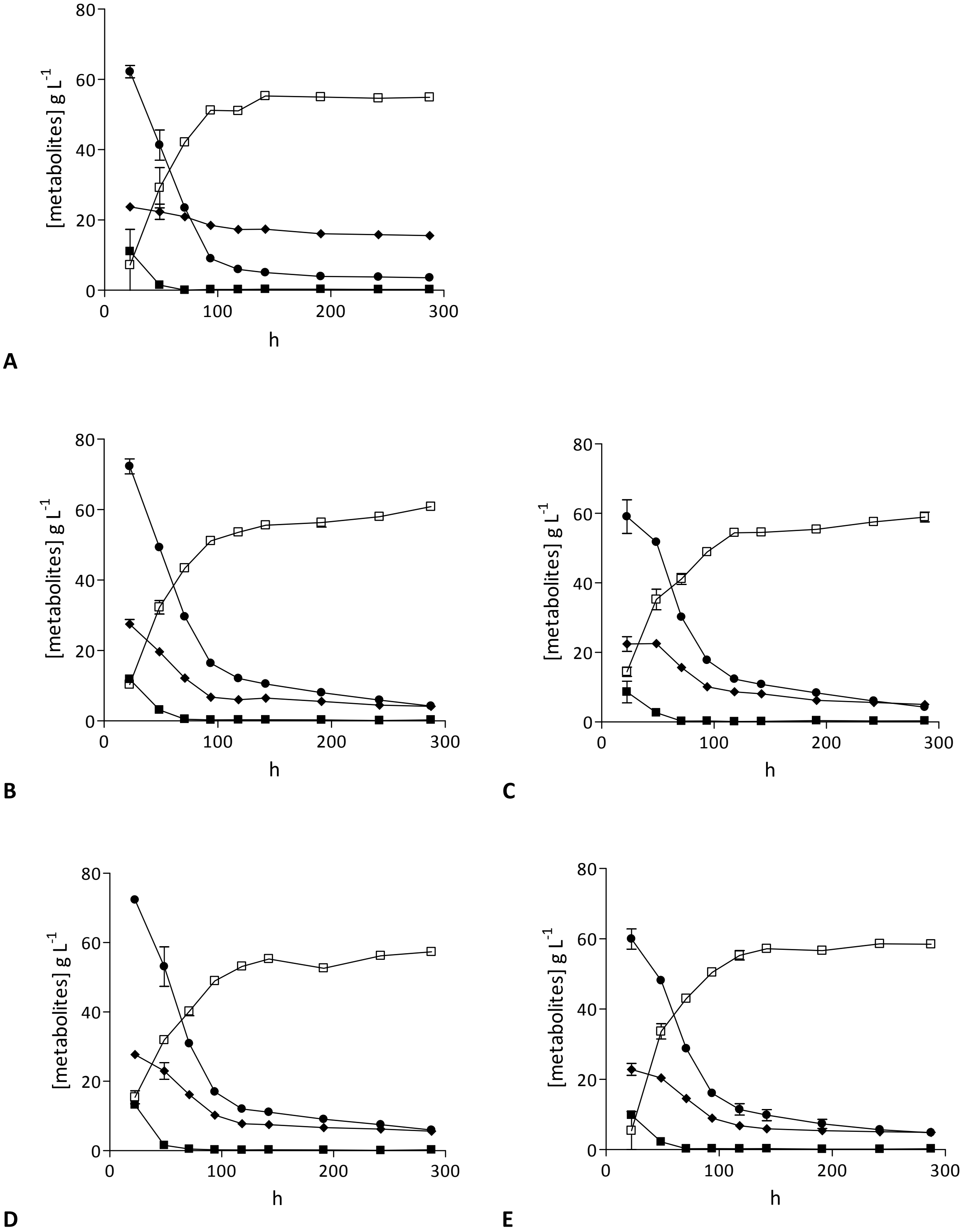

Figure S2

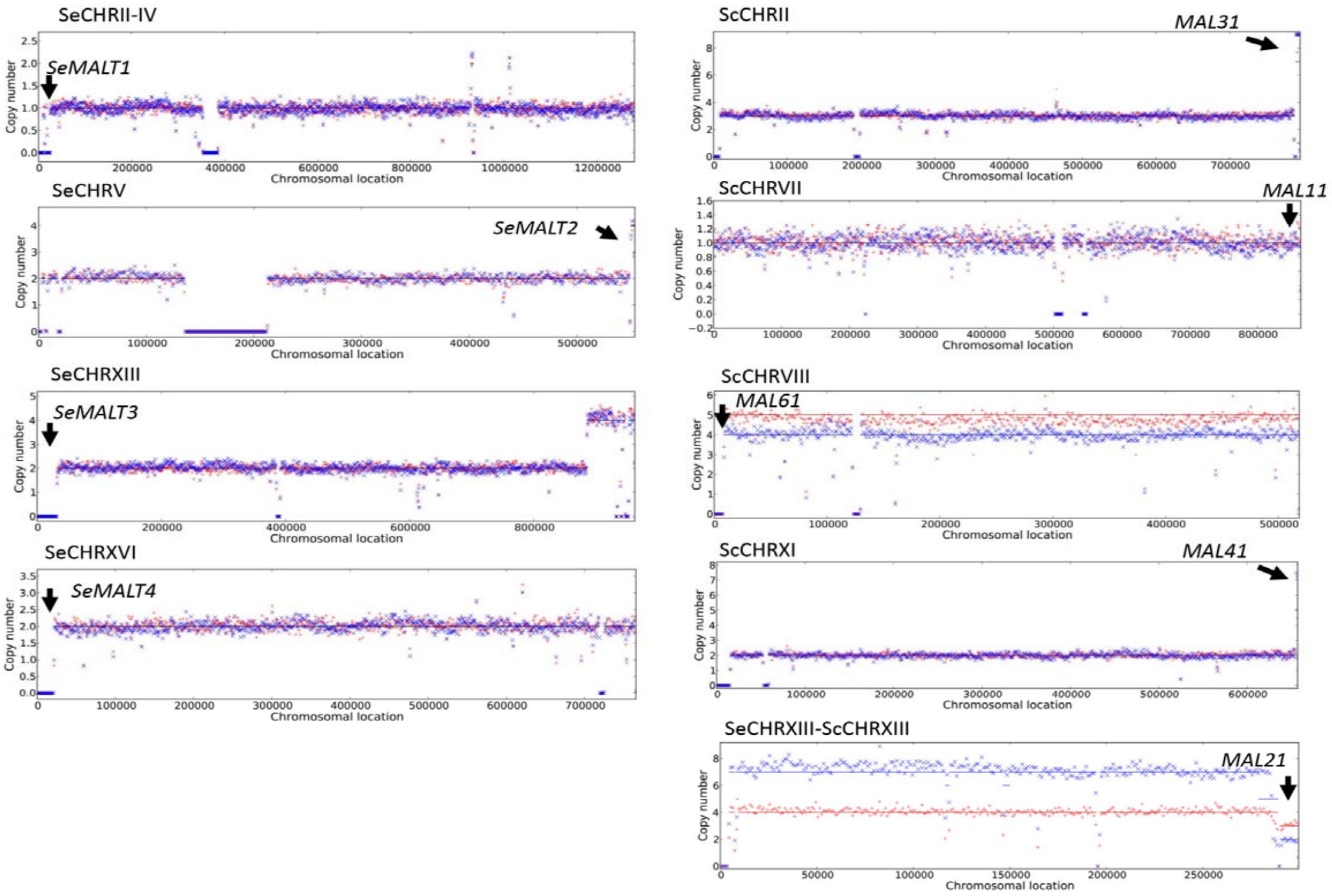
SeCHRII-IV
SeMALT1
SeCHRV
SeMALT2
SeCHRXIII
SeMALT3
SeCHRXVI
SeMALT4
ScCHRII
MAL31
ScCHRVII
MAL11
ScCHRVIII
MAL61
ScCHRXI
MAL41
SeCHRXIII-ScCHRXIII
MAL21
Copy number
Chromosomal location

Figure S3
